# Release activity-dependent control of vesicle endocytosis by the synaptic adhesion molecule N-cadherin

**DOI:** 10.1038/srep40865

**Published:** 2017-01-20

**Authors:** Bernd van Stegen, Sushma Dagar, Kurt Gottmann

**Affiliations:** 1 Institute of Neuro- and Sensory Physiology, Medical Faculty, Heinrich-Heine-University Düsseldorf, Universitätsstr. 1, 40225 Düsseldorf, Germany

## Abstract

At synapses in the mammalian brain, continuous information transfer requires the long-term maintenance of homeostatic coupling between exo- and endocytosis of synaptic vesicles. Because classical endocytosis is orders of magnitude slower than the millisecond-range exocytosis of vesicles, high frequency vesicle fusion could potentially compromise structural stability of synapses. However, the molecular mechanisms mediating the tight coupling of exo- and endocytosis are largely unknown. Here, we investigated the role of the transsynaptic adhesion molecules N-cadherin and Neuroligin1 in regulating vesicle exo- and endocytosis by using activity-induced FM4–64 staining and by using synaptophysin-pHluorin fluorescence imaging. The synaptic adhesion molecules N-cadherin and Neuroligin1 had distinct impacts on exo- and endocytosis at mature cortical synapses. Expression of Neuroligin1 enhanced vesicle release in a N-cadherin-dependent way. Most intriguingly, expression of N-cadherin enhanced both vesicle exo- and endocytosis. Further detailed analysis of N-cadherin knockout neurons revealed that the boosting of endocytosis by N-cadherin was largely dependent on preceding high levels of vesicle release activity. In summary, regulation of vesicle endocytosis was mediated at the molecular level by N-cadherin in a release activity-dependent manner. Because of its endocytosis enhancing function, N-cadherin might play an important role in the coupling of vesicle exo- and endocytosis.

At mature functional synapses, exo- as well as endocytosis of vesicles constitute the major subcellular events within the synaptic vesicle cycle^1^,^2^,^3^. A tight coupling of exocytotic vesicle fusion and endocytotic membrane retrieval is essential for stable maintenance of presynaptic membrane area and thus synapse size, even under conditions of high-frequency presynaptic activity^4^. However, how efficient exo-/endocytosis coupling is mediated at the molecular level is largely unknown.

Structural maintenance of complex pre- and postsynaptic domains has been proposed to be mediated by transsynaptic adhesion proteins coupled to scaffolding proteins and to the actin cytoskeleton. The transsynaptic N-cadherin adhesion system at excitatory, glutamatergic synapses is based on the homophilic interaction of pre- and postsynaptic N-cadherin^5^,^6^. N-cadherin signaling to the actin cytoskeleton is mediated by interaction with catenins thus forming the N-cadherin/catenin complex^7^,^8^. For the N-cadherin adhesion complex, postsynaptic roles in spine morphological differentiation, dynamics, and stabilization have been well established^9^,^10^,^11^,^12^,^13^,^14^. In contrast, potential roles of N-cadherin in modulating presynaptic vesicle cycling have remained unclear. N-cadherin has been suggested to be involved in synaptic vesicle clustering at nascent synapses^15^,^16^ and in modulating vesicle release at mature synapses^17^,^18^,^19^. In addition, postsynaptic N-cadherin has been proposed to influence vesicle release via interaction with presynaptic N-cadherin acting ultimately on actin filaments^17^,^18^,^19^,^20^.

In addition to homophilic systems, an essential heterophilic adhesion complex - the Neuroligin/Neurexin transsynaptic interaction - has been well described^21^,^22^,^23^,^24^. Neuroligin1 has been shown to exhibit synaptogenic effects in *in vitro* assays^25^,^26^,^27^, and to enhance vesicle release probability at mature synapses^28^,^29^. However, a role of transsynaptic adhesion systems in the regulation of vesicle endocytosis and in the coupling of exo- and endocytosis has hardly been investigated.

In this paper, we present evidence that N-cadherin signaling is involved in the control of synaptic vesicle endocytosis. Intriguingly, conditional knockout of N-cadherin led to a selective impairment of endocytosis that was induced by strong vesicle release activity. This suggests an important role of N-cadherin in regulating compensatory endocytosis in a strongly activity-dependent manner. Thus, the N-cadherin adhesion system might be a crucial molecular regulator of exo-/endocytosis coupling at mature central synapses.

## Results

### N-cadherin expression enhances both vesicle exo- and endocytosis at functional synapses

**Figure 1:**
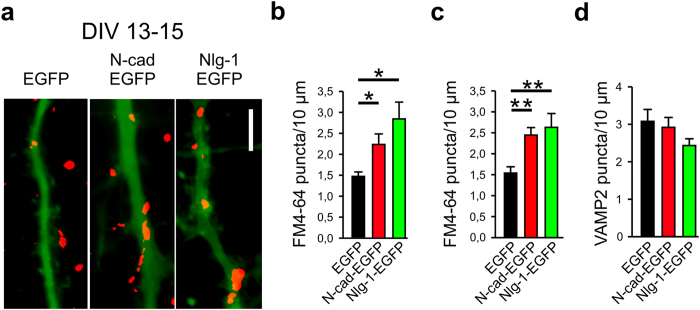
N-cadherin and neuroligin1 enhance vesicle cycling at mature synapses. (**a–d**) Overexpression of N-cadherin-EGFP (N-cad-EGFP) and Neuroligin1-EGFP (Nlg-1-EGFP), respectively, increased the dendritic density of FM4-64 labeled vesicle clusters (FM4-64 puncta) at 13–15 DIV, whereas the dendritic density of VAMP2 immunopositive puncta was unchanged. (**a**) Example overlay images of dendritic segments (green, EGFP fluorescence) and thresholded FM4-64 puncta (induced by electrical stimulation, red). Scale bar: 5 μm. (**b**) Quantification of dendritic density of FM4-64 puncta induced by K^+^ stimulation (90 mM). *n* (cells) = 26/15/18. (**c**) Quantification of dendritic density of FM4-64 puncta induced by electrical stimulation (400 AP-inducing stimuli at 20 Hz). *n* (cells) = 11/9/6. (**d**) Quantification of dendritic density of VAMP2 immunopositive puncta. *n* (cells) = 16/15/15. Means ± SEM are given. Statistical analysis was done using one-way ANOVA with Holm-Sidak posthoc test (**c**, P = 0.005 and P = 0.004), and Kruskal-Wallis one-way ANOVA on ranks with Dunn’s posthoc test (**b**, P < 0.05).

To begin to investigate the specific functional role of N-cadherin at mature synapses, we postsynaptically expressed a N-cadherin-EGFP fusion protein (transfection at 11 DIV) in individual cultured cortical neurons, and studied synaptic vesicle cycling (combined exo- and endocytosis) by FM4-64 staining of vesicle clusters at 13–15 DIV^30^. Analogous expression of Neuroligin1-EGFP was performed in parallel to enable comparison of N-cadherin effects to those of a well characterized synaptic adhesion molecule. Expression of both N-cadherin-EGFP and Neuroligin1-EGFP, respectively, did not result in an increase in the dendritic density of presynaptic, immunocytochemically stained VAMP2 puncta at 15 DIV (Fig. 1d, Suppl. Fig. 1). This demonstrates the absence of a synaptogenic effect after the major phase of *in vitro* synaptogenesis (see Suppl. Fig. 2 for synaptogenic effects at an earlier maturational stage (7 DIV)). Intriguingly, analysis at the functional level using stimulation-induced FM4-64 uptake revealed that expression of both N-cadherin- and Neuroligin1-EGFP fusion proteins, respectively, led to a strong increase in the dendritic density of FM4-64 puncta (Fig. 1a–c, Suppl. Fig. 1). This indicates a modulatory influence of postsynaptic N-cadherin on presynaptic cycling of vesicles at mature synapses, however, it was difficult to distinguish whether vesicle exocytosis or endocytosis or both were affected.

**Figure 2:**
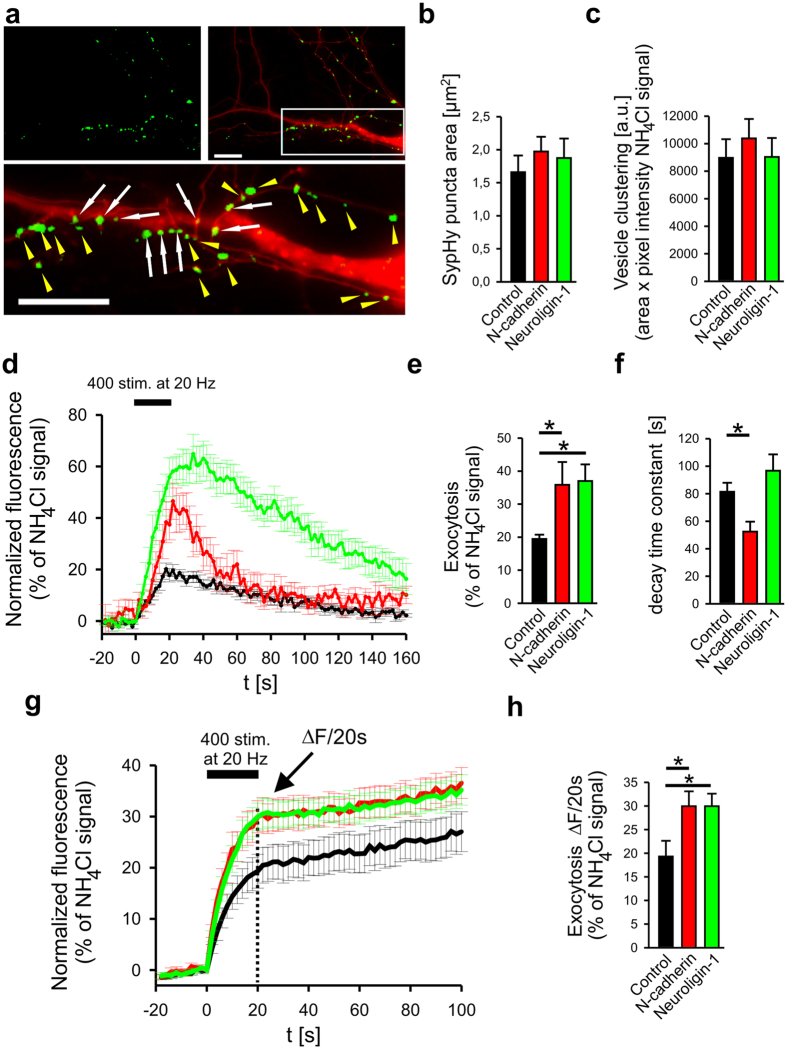
Analysis of the effects of N-cadherin and Neuroligin1 on synaptic exo- and endocytosis by synaptophysin-pHluorin (SypHy) fluorescence imaging. (**a**) Coexpression of SypHy and DsRed2 in individual cortical neurons cultured on glial microislands (analysed at 12–14 DIV). Upper panel, left: SypHy fluorescence (thresholded puncta, green) after 50 mM NH_4_Cl application to visualize vesicle clusters. Upper panel, right: overlay of SypHy (green) and DsRed2 (red) fluorescence to visualize vesicle cluster in the axon and on a proximal dendrite. Scale bar: 10 μm. Lower panel: distinction of SypHy puncta located on the proximal dendrite (white arrows) from non-contacting axonal vesicle clusters (yellow arrow heads). Boxed area is shown enlarged. Scale bar: 10 μm. (**b,c**) Quantification of autaptic (dendritic) SypHy puncta area (**b**) and total fluorescence intensity per autaptic punctum (**c**) upon expression of N-cadherin and Neuroligin1, respectively. (**d–f**) Effects of N-cadherin and Neuroligin1 expression on synaptic vesicle exo- and endocytosis. (**d**) Time course of SypHy fluorescence (normalized to NH_4_Cl signal of puncta) upon electrical stimulation (black bar). Signals from all autaptic (dendritic) SypHy puncta were averaged for each individual neuron expressing N-cadherin (red trace), Neuroligin1 (green trace), and for a control neuron (black trace). (**e,f**) Quantification of maximal amplitudes (**e**) and decay time constants (monoexponential fit, f) of stimulation induced SypHy signals. *n* (cells) = 10(55 autaptic puncta)/11(122 autaptic puncta)/8(60 autaptic puncta) (for **b,c** and **e,f**). (**g,h**) Effects of N-cadherin and Neuroligin1 expression on synaptic vesicle exocytosis in the presence of bafilomycin A1 (1 μM). (**g**) Time course of SypHy fluorescence (normalized to NH_4_Cl signal of puncta) upon electrical stimulation (black bar) in the presence of bafilomycin A1. (**h**) Quantification of stimulation induced exocytosis signal (SypHy fluorescence increase at the end of stimulation) in the presence of bafilomycin A1. *n* (cells) = 13/16/11. Means ± SEM are given. Statistical analysis was done using Kruskal-Wallis one-way ANOVA on ranks with Dunn´s posthoc test (**e**, P < 0.05; **h**, P < 0.05), and one-way ANOVA with Holm-Sidak posthoc test (**f**, P = 0.033).

To more specifically study the effects of N-cadherin expression on vesicle exo- and endocytosis, respectively, we monitored vesicle exo- and endocytosis using the pH-dependent fluorescence signal of synaptophysin-pHluorin (SypHy)^31^. SypHy was expressed in cortical neurons in autaptic microisland cultures, which enable simultaneous pre- and postsynaptic expression. Co-transfection (at 9 DIV) of SypHy and DsRed2 was used to visualize individual SypHy transfected neurons by DsRed2 fluorescence, and to identify autaptic vesicle clusters located on the dendrites of transfected neurons upon vesicular pH neutralization with 50 mM NH_4_Cl at the end of each experiment (Fig. 2a). At 12–14 DIV extracellular stimulation with 400 action potential-inducing stimuli at 20 Hz (for 20 sec) elicited punctate fluorescence increases due to exocytosis of synaptic vesicles (Fig. 2d; Suppl. Fig. 3a). Upon cessation of stimulation, this increase was followed by a slow (time constant ~80 sec at room temperature) decrease of fluorescence due to endocytosis and reacidification of synaptic vesicles. Exocytosis-induced SypHy fluorescence signals were strongly dependent on extracellular [Ca2^+^], and increased steadily with increasing extracellular [Ca2^+^] up to 5 mM (Suppl. Fig. 3).

At the maturational stage investigated neither expression of N-cadherin (without EGFP tag, co-transfected with SypHy and DsRed2) nor of Neuroligin1 (without EGFP tag) had synaptogenic effects as indicated by the unaltered mean area and fluorescence intensity of SypHy puncta revealed upon NH_4_Cl application at the end of each experiment (Fig. 2b,c). Furthermore, expression of N-cadherin led to a significantly enhanced exocytosis signal (Fig. 2d,e). Strikingly, N-cadherin expression resulted in addition in a significantly faster fluorescence decay indicating an enhanced endocytosis of synaptic vesicles (Fig. 2d,f). Expression of N-cadherin did not result in any significant change in reacidification kinetics as evident from experiments involving quenching of all surface SypHy molecules by a rapid pH step^31^,^32^ and then analysing the reacidification dependent SypHy fluorescence decay in endocytosed vesicles (Suppl. Fig. 4). In contrast, expression of Neuroligin1 did not lead to enhanced endocytosis, although it significantly increased exocytosis (Fig. 2d–f). The slight increase in decay time constant was not significant (Fig. 2f) and thus does not indicate a slow down of endocytosis.

To further confirm that expression of N-cadherin and Neuroligin1, respectively, results in enhanced exocytosis of synaptic vesicles, we performed SypHy fluorescence imaging as described above in the presence of the V-ATPase blocker bafilomycin A1 (1 μM) to block reacidification of endocytosed vesicles. It is well established that in the presence of bafilomycin A1 the exocytosis of synaptic vesicles can be quantitatively determined independent of vesicle endocytosis using pHluorin imaging^33^,^34^. We found that expression of both Neuroligin1 and N-cadherin significantly increased the amount of the stimulation-induced exocytosis signal (Fig. 2g,h), thus strongly corroborating enhancing effects of both synaptic adhesion molecules on the efficiacy of synaptic vesicle fusion. In summary, our results indicate that Neuroligin1 might directly enhance release probability by interacting transsynaptically at the active zone^28^, whereas N-cadherin might positively regulate exocytosis more indirectly. Most intriguingly, N-cadherin in addition strongly enhanced endocytosis of synaptic vesicles, thus enabling more efficient vesicle recycling to the active zone.

### N-cadherin deficiency results in impairment of vesicle endocytosis at high release activity

**Figure 3:**
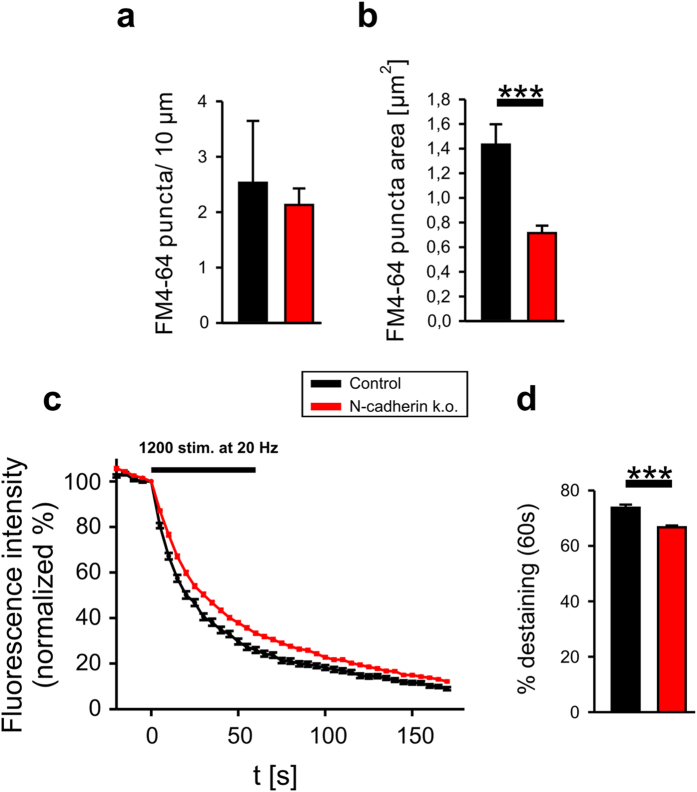
Conditional knockout of N-cadherin impairs vesicle cycling as indicated by FM4-64 staining/destaining. Cre (cre-EGFP vector cotransfected with EGFP) was expressed for 3–6 days in individual cortical neurons cultured from floxed N-cadherin mice. (**a**) Quantification of dendritic density (per 10 μm dendrite length) of FM4-64 puncta (stained with 400 AP-inducing stimuli at 20 Hz at 12–15 DIV). *n* (cells) = 4/6. (**b**) Quantification of FM4-64 puncta area indicating reduced vesicle endocytosis upon knockout of N-cadherin in the postsynaptic neuron. *n* (puncta on postsynaptic dendrites) = 82/105. (**c**) FM 4–64 destaining kinetics upon electrical stimulation with 1200 AP-inducing stimuli at 20 Hz (black bar). Destaining kinetics of individual puncta are averaged. *n* (puncta on postsynaptic dendrites) = 91/165. Note the slower destaining upon knockout of N-cadherin. (**d**) Quantification of the amount of destaining per punctum at the end of stimulation (after 60 seconds). Means ± SEM are given. Statistical analysis was done using Student’s t-test (**b,d**, P < 0.001).

**Figure 4:**
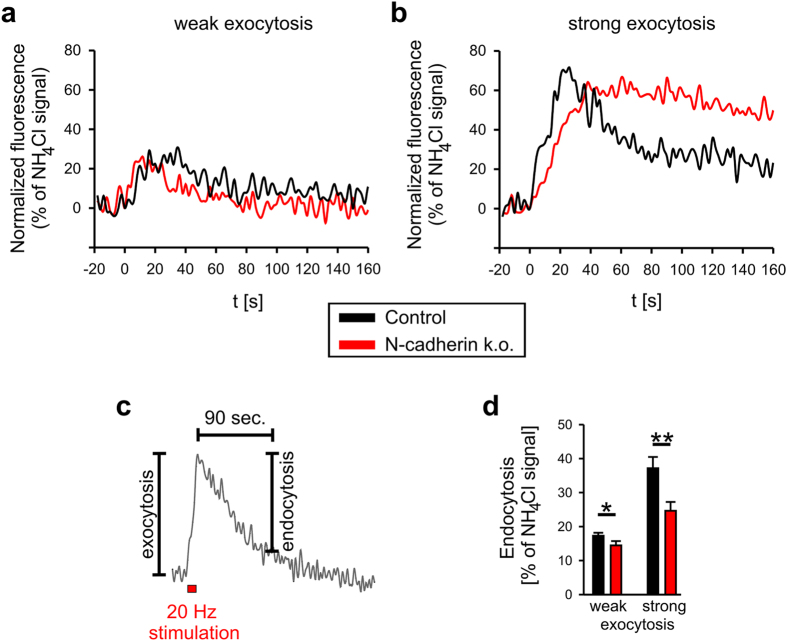
Amount of exocytosis correlates with N-cadherin dependence of endocytosis. (**a,b**) Upon strong exocytosis, the inhibition of endocytosis by conditional N-cadherin knockout (by Cre expression in individual cortical neurons in culture) is more pronounced. Examples of stimulation-induced SypHy fluorescence changes (determined at individual synaptic puncta) from control (black traces) and N-cadherin knockout (red traces) neurons at weak (**a**) and strong (**b**) exocytosis (13–14 DIV). (**c**) Scheme of quantification of endocytosis (90 sec after end of stimulation). (**d**) Quantification of endocytosis upon weak (≤30% NH_4_Cl signal) and strong (≥60% NH_4_Cl signal) exocytosis in control (black bars) and N-cadherin knockout (red bars) neurons. Stimulation protocols consisting of 200 up to 800 stimulations at 20 Hz in 2 mM or 5 mM Ca2+ (for 10 to 40 sec) were used to obtain weak and strong exocytosis signals. *n* (autaptic puncta responding to stimulation) = 46 from 19 cells and 41 from 10 cells (weak exocytosis)/10 from 7 cells and 9 from 6 cells (strong exocytosis). Means ± SEM are given. Statistical analysis was done using Student’s t-test (**d**, P = 0.026 (weak exocytosis), P = 0.009 (strong exocytosis)).

**Figure 5:**
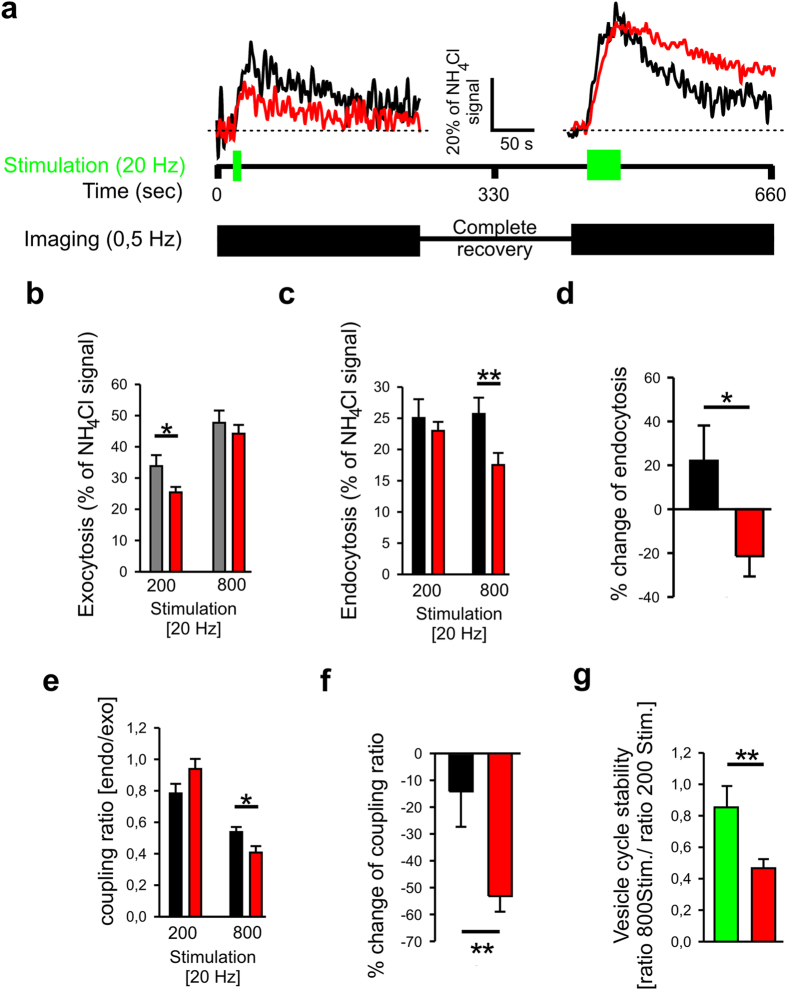
N-cadherin regulation of endocytosis depends on preceding release activity. Comparison of SypHy fluorescence signals upon successive weak and strong stimulation in individual neurons. (**a**) Example SypHy signals obtained during successive weak (200 stimuli, left) and strong (800 stimuli, right) stimulation in a control (black traces) and a N-cadherin knockout (red traces) neuron. (**b,c**) Quantification (see Fig. 4c) of mean exocytosis (**b**) and mean endocytosis (**c**) with 200 and 800 stimuli, respectively. (**d**) Comparison of endocytosis in individual SypHy puncta by calculating the %change of endocytosis at successive weak and strong stimulation. (**e,f**) Quantification of mean coupling ratio (endocytosis/exocytosis) (**e**) and comparison of coupling ratio in individual SypHy puncta (**f**). (**g**) Vesicle cycle stability (coupling ratio 800stim./coupling ratio 200stim.). Gray, black and green bars, respectively, represent data from control neurons. Red bars represent data from conditional N-cadherin knockout neurons. *n* (autaptic puncta responding to double stimulation) = 22 from 11 cells for control, and 25 from 5 cells for N-cadherin knockout. Note the reduction in endocytosis at strong stimulation in N-cadherin knockout neurons. Means ± SEM are given. Statistical analysis was done using Student’s t-test (**b**, P = 0.040; **c**, P = 0.009; **e**, P = 0.016; **g**, P = 0.007), and Mann-Whitney Rank Sum test (**d**, P = 0.015; **f**, P = 0.004).

To further study the role of N-cadherin in regulating vesicle endocytosis, we performed a conditional knockout of N-cadherin in individual cortical neurons in cultures from floxed N-cadherin mice^35^ by sparse expression of Cre^36^,^37^ (transfection at 9 DIV, analysis of postsynaptic N-cadherin deficient neurons at 12–15 DIV). As characterized previously^37^, loss of N-cadherin at the protein level requires several days following Cre transfection. First, we studied vesicle recycling in Cre-transfected neurons by extracellular stimulation-induced (400 stimuli at 20 Hz) uptake of FM4-64 (10 μM) in vesicles leading to fluorescent FM4-64 puncta on dendrites. Intriguingly, we observed a strongly reduced area of FM4-64 puncta in N-cadherin deficient neurons (Fig. 3a,b), which is in line with an impairment of vesicle endocytosis at synapses, but does not exclude that this is alternatively caused by altered vesicle pool size or altered exocytosis. Therefore, additional experiments using SypHy fluorescence imaging were required to establish an alteration in endocytosis in N-cadherin deficient neurons (see Figs 4 and 5). In addition, extracellular stimulation-induced (1200 stimuli at 20 Hz) destaining of FM4-64 puncta revealed a slightly reduced vesicle release in N-cadherin deficient synapses (Fig. 3c,d). Moreover, Cre-induced N-cadherin knockout led to a strong inhibition of the Neuroligin1-induced increase in vesicle cycling due to impaired Neuroligin1 synaptic recruitment (Suppl. Fig. 5). This is confirming a previous paper by our group that described a N-cadherin requirement for the enhancing effect of Neuroligin1 overexpression on exocytosis^16^. Together, these data strongly indicate N-cadherin protein loss upon Cre expression at the functional level. In summary, our loss-of-function approach strongly supported an important role of N-cadherin in synaptic vesicle endocytosis.

To further investigate the role of N-cadherin in the regulation of endocytosis by using SypHy fluorescence imaging, N-cadherin knockout was again induced in cortical neurons from floxed N-cadherin mice by Cre expression in individual neurons grown in autaptic microisland cultures. Co-transfection of cre, SypHy and DsRed2 was done at 9 DIV, and SypHy fluorescence imaging was performed at 13–14 DIV to allow for turnover of N-cadherin protein present prior to induction of gene knockout^37^. To study the release dependence of endocytosis regulation by N-cadherin, SypHy fluorescence signals from individual puncta showing weak (exocytosis range 10–30% of NH_4_Cl signal) and strong (60–80% of NH_4_Cl signal) exocytosis were compared (Fig. 4a,b). Because of the relatively high noise levels of SypHy signals from individual puncta, endocytosis was quantitatively determined relative to the NH_4_Cl-induced signal (Fig. 4c) instead of fitting their decay time course. At weak exocytosis, N-cadherin knockout resulted only in a very slight reduction of endocytosis indicating that N-cadherin may not be required for this mode of endocytosis. At strong exocytosis, endocytosis was increased and now strongly dependent on the presence of N-cadherin suggesting the existence of a second, strongly N-cadherin dependent mode of endocytosis (Fig. 4c,d).

To more directly study the release dependence of endocytosis control by N-cadherin, we studied SypHy fluorescence signals elicited by two different stimulation paradigms applied in succession to the same neuron (co-transfected with cre, SypHy, DsRed2 in microisland cultures as described above). The first stimulus consisted of 200 action potentials at 20 Hz (weak exocytosis), whereas the second stimulus consisted of 800 action potentials at 20 Hz resulting in an increased exocytosis signal (Fig. 5a). As expected from the FM destaining experiments, knockout of N-cadherin resulted in a slightly reduced exocytosis (with 200 APs at 20 Hz; Fig. 5b). A slight, but non significant decrease in exocytosis comparable in size to the reduction of exocytosis in FM destaining experiments (Fig. 3d) was also found with stronger stimulation (800 APs at 20 Hz). Strikingly, endocytosis was affected differentially in N-cadherin deficient neurons. Under weak stimulation endocytosis was unaffected, whereas with strong stimulation endocytosis was significantly inhibited (Fig. 5c). Direct comparison of the SypHy signals obtained from individual puncta revealed an increase in endocytosis at the second stimulation in control neurons, whereas endocytosis was decreased in N-cadherin deficient neurons, resulting in a significant difference (Fig. 5d). Similarly, the coupling ratio (% endocytosis/% exocytosis) was unaffected under weak stimulation, but was significantly reduced with strong stimulation in N-cadherin deficient neurons (Fig. 5e). Direct comparison of the SypHy signals obtained from individual puncta revealed only a very slight change in the coupling ratio at the second stimulation in control neurons, whereas coupling ratio was strongly decreased in N-cadherin deficient neurons, again resulting in a significant difference (Fig. 5f). Furthermore, in the presence of N-cadherin the coupling ratio was similar at 200 and 800 stimuli (i.e. high vesicle cycle stability), whereas vesicle cycle stability was strongly decreased in N-cadherin deficient neurons (Fig. 5g). In summary, our results strongly indicate that N-cadherin is not essential for a mode of endocytosis elicited by relatively weak vesicle release, whereas it is crucially involved in compensatory endocytosis elicited by strong vesicle release.

## Discussion

In this paper, we present first evidence that the synaptic adhesion molecule N-cadherin might play a central role in the homeostatic coupling of exo- and endocytosis at highly active cortical excitatory synapses. As indicated by gain- and loss-of-function approaches, N-cadherin enhances vesicle endocytosis rate upon strong vesicle release. In addition, N-cadherin also modulated exocytosis of synaptic vesicles, most likely as a consequence of its endocytosis promoting effect.

A modulatory influence of N-cadherin on endocytosis has been proposed previously on the basis of expression of dominant-negative mutant N-cadherin followed by FM staining/destaining^9^,^19^. However, FM dye uptake is dependent on both vesicle exo- and endocytosis, thus making it difficult to conclude which part of the synaptic vesicle cycle is affected. No direct examination of vesicle endocytosis using pHluorin-based imaging has yet been performed. In our SypHy imaging experiments, expression of N-cadherin resulted in an enhanced vesicle endocytosis rate. This was not simply caused by the in parallel observed increase in exocytosis, because control experiments using Neuroligin1 overexpression resulted in a similar increased exocytosis, but did not lead to altered endocytosis. In addition, our N-cadherin knockout approach revealed an endocytosis promoting function that was most pronounced after strong vesicle release. Taken together, these gain- and loss-of-function approaches indicate that N-cadherin plays an important role in enhancing compensatory endocytosis after substantial addition of vesicle membrane to the presynaptic membrane. In line with a role of N-cadherin in promoting vesicle endocytosis, N-cadherin has been described to localize to the peri-active zone at mature glutamatergic synapses (i.e. adjacent to, but largely outside of the active zone)^38^,^39^, and thus close to the proposed endocytosis zone of mature presynaptic boutons^40^. Regarding the molecular mechanism, N-cadherin might exert its enhancing effect by activating a catenin-dependent reorganization of actin filaments^41^,^42^ that might lead to altered myosin-dependent translocation of vesicles^43^.

Moreover, a modulatory role of N-cadherin in vesicle exocytosis has been proposed based on the expression of dominant-negative N-cadherin mutant proteins^17^,^19^. In addition, vesicle release-induced FM destaining upon high-frequency stimulation was impaired in N-cadherin knockout neurons^18^ (see also Fig. 3c). In this paper, a modulatory influence of N-cadherin on vesicle exocytosis was indicated in both overexpression and knockout experiments. In particular, in SypHy imaging experiments involving block of reacidification of re-endocytosed vesicles by bafilomycin A1 an enhancing effect of N-cadherin on exocytosis in the same range as the well known enhancing effect of Neuroligin1 on release probability^20^,^21^,^22^,^28^,^29^ was clearly evident. However, the mechanisms involved are incompletely understood. Because of the well established peri-active zone localization of N-cadherin at mature glutamatergic synapses^38^,^39^, a highly indirect action on exocytosis appears mandatory. A N-cadherin dependent facilitation of vesicle endocytosis that leads to a more efficient vesicle replenishment at the active zone via the presynaptic vesicle cycle might well account for the observed modulation of vesicle exocytosis by N-cadherin. In contrast, other synaptic adhesion molecules, e.g. the Neuroligin1/Neurexin transsynaptic system, appear to affect vesicle exocytosis rather directly without any influence on endocytosis.

In summary, we obtained evidence for a central role of N-cadherin in controling the coupling of exo- and endocytosis at mature synapses under conditions of strong vesicle release. In this cellular context, N-cadherin function is essential for triggering compensatory endocytosis thus balancing vesicle membrane addition and stabilizing the presynapse.

## Methods

Methods were carried out in accordance with the relevant guidelines.

### Cell culture

Mass cultures of mouse cortical neurons were done as described previously^16^,^44^,^45^. In brief, cortices of E18-19 mouse fetuses from C57/BL6 wildtype mice and from Ncad^flox/flox^ mice^35^, respectively, were prepared and mechanically dissociated after 5 min trypsin treatment. The method of sacrifying mice was approved by the Tierschutzbeauftragte of the University of Düsseldorf. 30.000–40.000 cells were seeded on poly-L-ornithine (1 mg/ml; Sigma-Aldrich) coated coverslips for 1 hour at 37 °C. Further cultivation (for up to 15 days) was done in 2 ml Neurobasal (NB) medium (Gibco) with B27 supplement (2%, 50x; Gibco), Glutamax-I supplement (Gibco), penicilline-streptomycine (Gibco) added.

Autaptic glial microisland cultures enabling both pre- and postsynaptic expression simultaneously were done as described previously^46^. Confluent monolayers of mouse astrocytes were obtained from E18/19 or P0-P3 cortical tissue from C57/BL6 wildtype mice by dissociation and long-term cultivation in BME medium containing 10% FBS. For glial microisland cultures astrocytes were detached from culture flasks, seeded at low density on glass coverslips, and cultured for 4–7 days in BME/10% FBS medium. Then, 40.000–80.000 disscociated cortical neurons (see above) were added per coverslip and after 6 h BME/10%FBS medium was replaced by NB medium with B27 supplement for further cultivation. Culture medium was exchanged every 3 days.

### Transfection and plasmids

Sparse transfection of individual cultured neurons was done using a magnetofection procedure based on magnetic nanoparticles^47^. Plasmid DNA was added to magnetic nanoparticles (NeuroMag; OZ Biosciences) in NB medium without supplements and incubated for 15 min at room temperature. DNA/NeuroMag-complexes were added to cell cultures using 6-well plates for 30 min at 37 °C and transfection efficiency was enhanced by applying an oscillating magnetic field (Magnetofection^TM^, magnefect LT; nanoTherics). After transfection the culture medium was replaced with fresh medium for further culture. For conditional knockout of N-cadherin, cells cultured from N-cadherin^flox/flox^ mice^35^ (from Jackson Labs) were cotransfected with an EGFPcre plasmid^36^ (from Addgene).

The following plasmids were used: pEGFP-N1 (Clontech), pDsRed2-N1 (Clontech), Neuroligin-1-EGFP (gift from Dr. T. Dresbach, Göttingen, Germany), pCMV-Neuroligin-1 (gift from Dr. N. Brose, Göttingen, Germany), N-cadherin-EGFP (gift from Dr. C. Gauthier-Rouviere, Montpellier, France), pMS149.1-N-cadherin (gift from Dr. R. Kemler, Freiburg, Germany), SypHy-A4 (gift from Dr. L. Lagnado, Cambridge, UK), pBS598EF1alpha-EGFPcre (Addgene). The Neuroligin1-EGFP, pCMV-Neuroligin-1, N-cadherin-EGFP, pMS149.1-N-cadherin plasmids were sequenced during their use for this paper.

In initial experiments (FM 4–64 staining/destaining, Fig. 1) sparse DNA transfection of cultured neurons was performed by using Lipofectamine 2000 (Invitrogen) following the manufacturer’s instructions as described previously^16^.

### Immunocytochemistry and antibodies

Immunocytochemical stainings were performed as described previously^16^,^37^. The following primary antibodies were used: Anti-VAMP2 (rabbit polyclonal, 1:2000, Abcam Cat. No. 3347); Anti-N-cadherin (rabbit polyclonal, 1:500–1:2000, Abcam Cat. No. 18203); Anti-PSD95 (mouse monoclonal, 1:400–1:1000, Abcam Cat. No. 2723); Anti-VAMP2 (guinea pig polyclonal, 1:500, Synaptic Systems Cat. No. 104204).

### Fluorescence imaging and data analysis

Wide-field fluorescence imaging was performed using an inverted motorized Axiovert 200 M microscope (Zeiss) as described previously^16^,^37^. In brief, images were obtained with a 12 bit CoolSnap ES2 CCD camera (Photometrics). For immunocytochemistry, 3D z-stacks were done at 0.3 to 0.5 μm depth using MetaVue software (Molecular Devices/Visitron Systems). The following filter sets (Zeiss) were used: i) excitation 365 nm, beam splitter 395 nm, emission 445/50 nm (DAPI); ii) excitation 485/20 nm, beam splitter 510 nm, emission 515/565 nm (FITC; EGFP, SypHy); iii) excitation 545/25 nm, beam splitter 570 nm, emission 605/70 nm (CY3; FM4–64, DsRed2).

For data analysis, z-stacks of images were further processed offline with AutoDeblur software (Visitron Systems) for deconvolution and thereby correction of optical distortions as described previously^16^. Deconvolved z-stacks were further processed using Metamorph software (Molecular Devices/Visitron Systems) to obtain maximum projection images that were thresholded. If necessary, thresholded images were low-pass filtered to exclude single pixel noise prior to quantitative analyses of user-defined regions-of-interest (ROIs) using Metamorph software. From ROIs average pixel intensity, puncta area and puncta density were determined and further analysed using SigmaPlot 11 software (Systat).

### Electrical stimulation

Extracellular field stimulation (for FM4-64 and SypHy imaging) was done using a stimulation chamber (Chamlide, Live Cell Instrument) on the stage of an Axiovert 200 M (Zeiss) microscope. 1 ms biphasic pulses of 100 mA (A385/A382 World Precision Instruments) at 20 Hz were used.

### FM4-64 staining/destaining and data analysis

To label presynaptic vesicle clusters, staining of recycling synaptic vesicles was performed using the styryl dye FM4-64 according to standard protocols at room temperature^30^,^48^. For dye uptake into cultured cortical neurons, FM4-64 (10 μM) was added to the extracellular solution (containing in mM 119 NaCl, 2.5 KCl, 2 CaCl_2_, 2 MgCl_2_, 25 HEPES, 30 glucose, pH = 7.4 with addition of 50 μM DL-AP5 and 10 μM DNQX) and cultures were stimulated either by electrical stimulation (400 action potential-inducing stimuli at 20 Hz) or by increasing extracellular [K^+^] in the extracellular solution (containing in mM 45 NaCl, 90 KCl, 2.5 CaCl_2_, 1 MgCl_2_, 20 HEPES, pH = 7.3) for 2 min. 60 sec after stimulation, FM4-64 dye was removed from the surface membrane by a 10 min wash in Ca^2+^-free extracellular solution with addition of 1 mM ADVASEP-7 and then fluorescent FM4-64 puncta were imaged. For studying exocytosis, destaining of FM4-64 labeled vesicle clusters was elicited by electrical stimulation in extracellular solution (1200 action potential-inducing stimuli at 20 Hz for 60 sec). Fluorescence images were taken every 5 sec during destaining.

Quantitative analysis of FM4-64 staining/destaing experiments was done using MetaMorph/MetaVue software (Molecular Devices/Visitron Systems) as described previously^16^,^48^. In brief, FM4-64 fluorescence images were thresholded according to background levels^16^ and overlayed on images of EGFP (or EGFP fusion protein) expressing dendrites to identify FM4-64 puncta on dendrites of transfected neurons. Regions of interest (ROI) were created around puncta for quantitative analysis of individual puncta. Image sequences from destaining experiments were analysed by creating ROIs in a similar way and by determining fluorescence intensites over time for each punctum after background subtraction. Decay time constants were determined by monoexponential fitting the mean fluorescence decay obtained by averaging the normalized destaining curves of individual FM4-64 puncta for each cell using Sigmaplot 11 software (Systat).

### Synaptophysin-pHluorin (SypHy) imaging and data analysis

To study endocytosis of synaptic vesicles quantitatively, the pH sensitive fluorescent probe synaptophysin-pHluorin (SypHy)^31^ was used according to standard protocols^49^,^50^. In brief, cortical neurons in autaptic microisland cultures were cotransfected with SypHy-A4 and DsRed2 (and specific adhesion molecules) to visualize transfected neurons and their dendrites. Experiments were performed 3 to 5 days after transfection at room temperature.

To induce vesicle exocytosis, cultures were transfered to a stimulation chamber (Live Cell Instruments) on the stage of an Axiovert 200 M (Zeiss) microscope, and were electrically stimulated in extracellular solution containing in mM 136 NaCl, 2.5 KCl, 2 CaCl_2_, 1.3 MgCl_2_, 10 HEPES, 10 glucose, pH = 7.3 with 50 μM DL-AP5 and 10 μM DNQX added to prevent recurrent network activity. At the end of the experiment, vesicles were alkalized by incubating cultures in an extracellular solution containing in mM 50 NH_4_Cl, 86 NaCl, 2.5 KCl, 2 CaCl_2_, 1.3 MgCl_2_, 10 HEPES, 10 glucose, pH = 7.3 to obtain the maximal SypHy fluorescence. During and after stimulation, fluorescence images were acquired at time intervals of 2–5 sec to obtain SypHy fluorescence transients.

SypHy fluorescence intensities over time were analysed offline using MetaVue software. ROIs were defined in transfected neurons around autaptic contacts, which were identified by thresholding the fluorescence image obtained in NH_4_Cl containing extracellular solution according to background levels and overlaying it onto the corresponding DsRed2 fluorescence image of dendrites. For quantitative analysis of fluorescence transients, the measured average fluorescence intensity within a ROI was first background subtracted at each time point using SigmaPlot 11 software. Then baseline intensity was subtracted to compensate for differences in spontaneous activity. Finally, the intensity values were normalized to the maximal fluorescence signal (background subtracted) obtained in NH_4_Cl containing extracellular solution at the end of the experiment. Puncta not showing a fluorescence increase upon stimulation (non-releasing sites) were excluded from analysis. In double-stimulation experiments, puncta not showing an increased SypHy fluorescence signal upon the second stronger stimulation were excluded from analysis, because of disturbance of vesicle cycling during the experiment.

### Statistics

All data are given as means ± SEM. Statistical significance was determined by one-way ANOVA in combination with the Holm-Sidak posthoc test by using SigmaPlot 11 software (Systat). Normality of data was tested using SigmaPlot 11 software. If this test failed, ANOVA on ranks was used (see figure legends). For statistical comparison of no more than two experimental groups Student’s t-test was performed by using SigmaPlot 11 software.

## Additional Information

**How to cite this article**: van Stegen, B. *et al*. Release activity-dependent control of vesicle endocytosis by the synaptic adhesion molecule N-cadherin. *Sci. Rep.* **7**, 40865; doi: 10.1038/srep40865 (2017).

**Publisher's note:** Springer Nature remains neutral with regard to jurisdictional claims in published maps and institutional affiliations.

## Supplementary Material

Supplementary Figures
