# Supplementary material for: Release activity-dependent control of vesicle endocytosis by the synaptic adhesion molecule N-cadherin
Source: Sci Rep. 2017 Jan 20;7:40865. doi: 10.1038/srep40865 (PMC5247765; doi:10.1038/srep40865)
Supplement: Supplementary Figures [file srep40865-s1.pdf]

**Supplementary figures:** Bernd van Stegen, Sushma Dagar, and Kurt Gottmann. Release activity-dependent control of vesicle endocytosis by the synaptic adhesion molecule N-cadherin

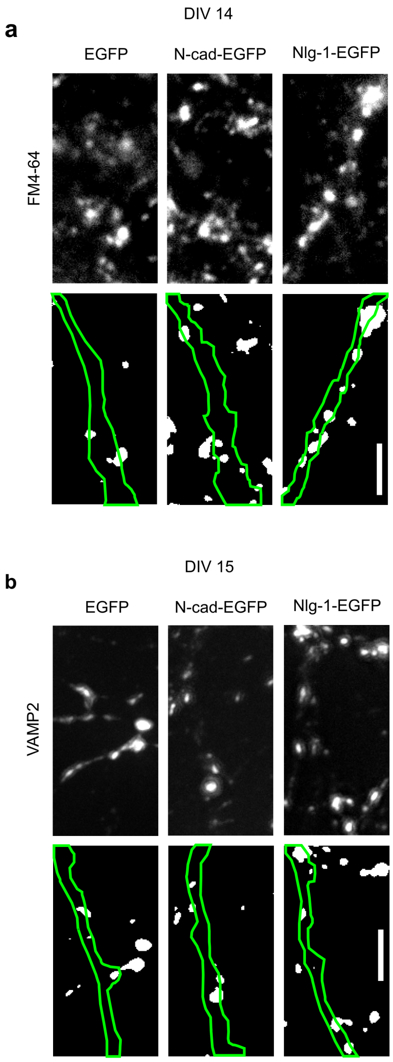


**Suppl. Figure 1. N-cadherin-EGFP and Neuroligin1-EGFP expression (for 3 days) at 14-15 DIV, respectively, increased FM4-64 puncta density, whereas VAMP2 puncta density was unaffected (absence of synaptogenic activity).** (**a**) $K^+$ stimulation (90 mM) induced FM4-64 puncta (upper panel) on dendrites (outlined green; lower panel with FM4-64 puncta thresholded). Scale bar: 5 µm. (**b**) Immunostained VAMP2 puncta (upper panel) on dendrites (outlined green; lower panel with VAMP2 puncta thresholded). Scale bar: 5 µm.

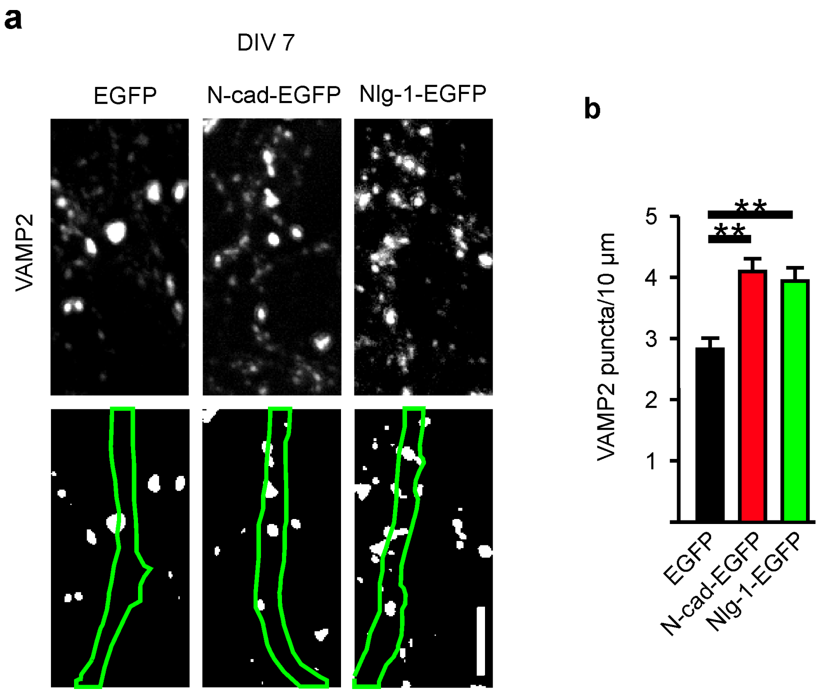


**Suppl. Figure 2. Synaptogenic activity of Neuroligin1-EGFP and N-cadherin-EGFP upon expression (for 3 days) in immature neurons at 7 DIV.** (**a**) Immunostained VAMP2 puncta (upper panel) on dendrites (outlined green; lower panel with VAMP2 puncta thresholded). Scale bar: 5 µm. (**b**) Quantification of dendritic density (per 10 µm dendrite length) of VAMP2 puncta. *n* (cells) = 29 / 29 / 25. Means ± SEM. One-way ANOVA with Holm-Sidak posthoc test (b, $P<0.001$).

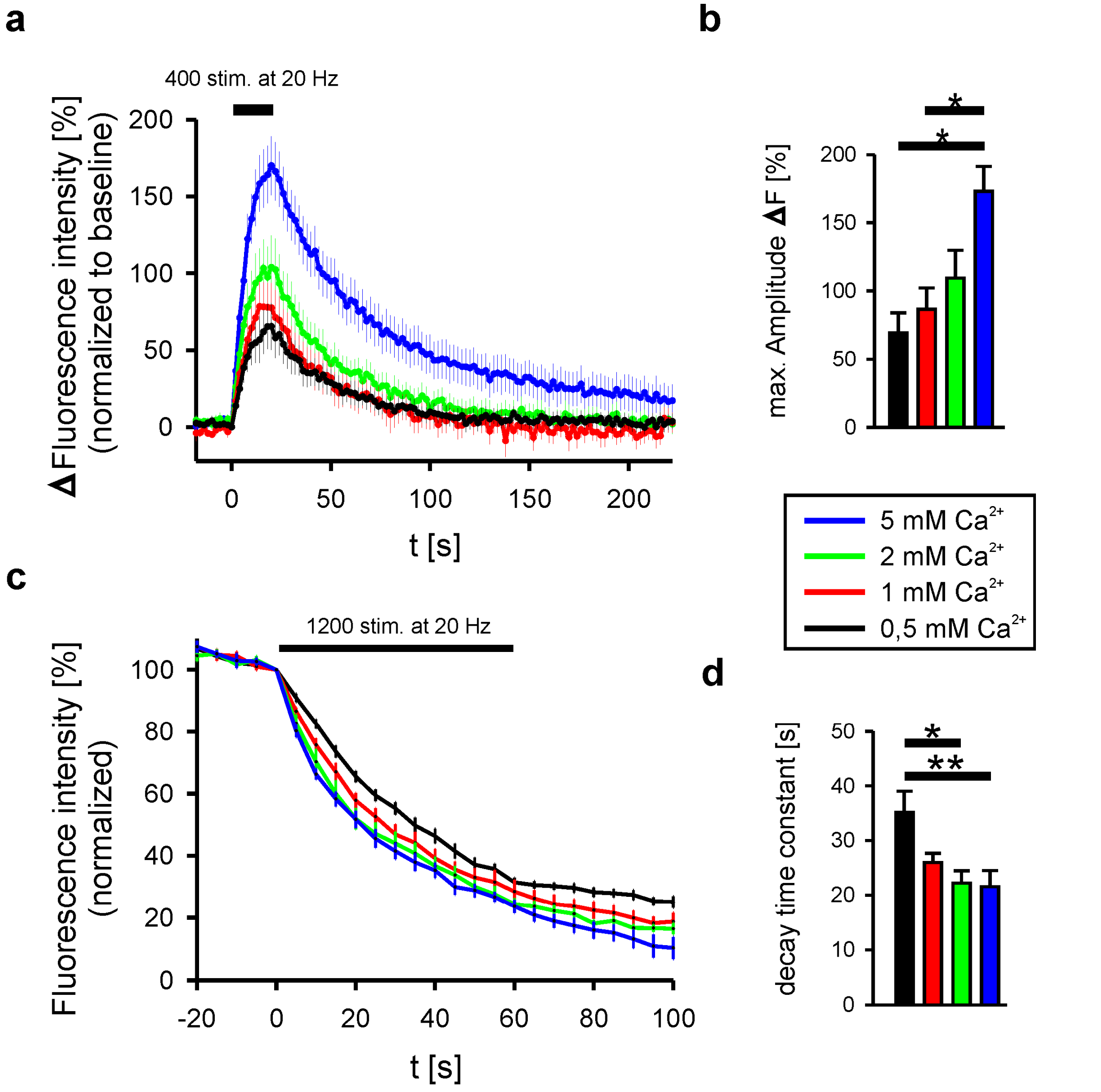


**Suppl. Figure 3. Differential sensitivity of SypHy fluorescence imaging and FM4-64 destaining kinetics for extracellular [$Ca^{2+}$] induced changes in vesicle release.** (**a**) SypHy fluorescence signals induced by electrical stimulation at different extracellular $Ca^{2+}$ concentrations as indicated (colour code inset). Normalized signals from individual puncta were averaged for each cell and then cells were averaged. (**b**) Quantification of peak amplitudes of SypHy signals (representing vesicle exocytosis) at different extracellular [$Ca^{2+}$]. *n* (cells) = 9 / 8 / 7 / 9. (**c**) FM4-64 destaining kinetics (induced by electrical stimulation) at different extracellular $Ca^{2+}$ concentrations as indicated. Normalized signals from individual puncta were averaged for each cell and then cells were averaged. (**d**) Quantification of FM4-64 decay time constants at different extracellular [$Ca^{2+}$]. *n* (cells) = 8 / 12 / 8 / 9. Means ± SEM. Kruskal-Wallis one-way ANOVA on ranks with Dunn´s posthoc test (b, $P<0.05$; c, $P<0.05$, $P<0.01$).

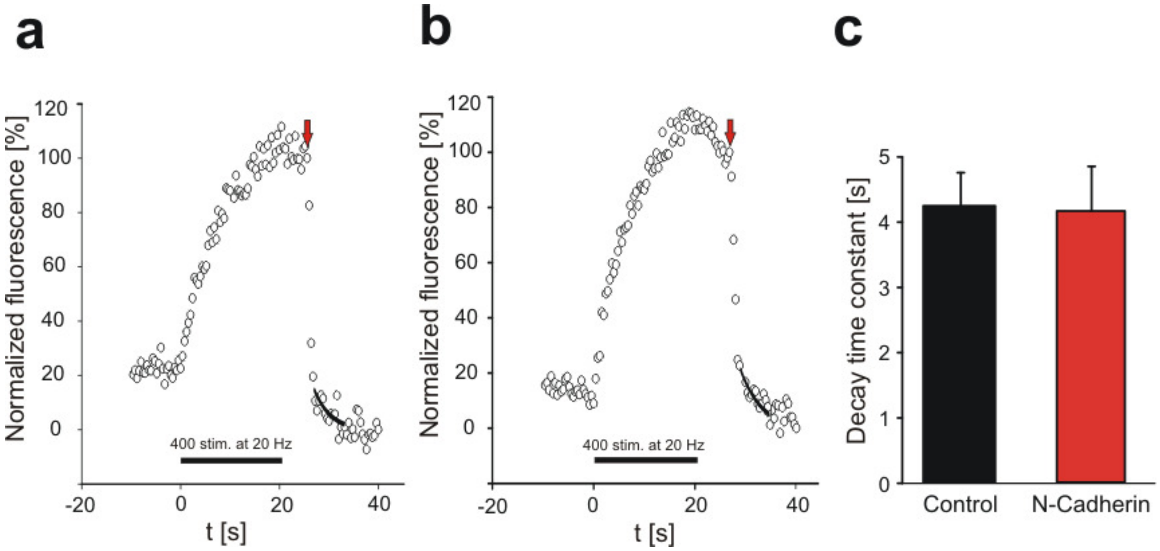


**Suppl. Figure 4. Expression of N-cadherin does not alter reacidification kinetics in endocytosed vesicles.** (**a, b**) Example traces of SypHy imaging experiments designed to determine reacidification kinetics (individual puncta averaged). After electrical stimulation (400 stimuli at 20 Hz) to induce vesicle fusion, a rapid extracellular pH drop (red arrows, final pH 5.25) was performed to quench the fluorescence of cell surface SypHy molecules. Control experiments in the presence of dynasore (80 µM, n=3) revealed a mean duration of the pH step of 1.47 ± 0.46 sec. SypHy molecules in vesicles endocytosed before the pH step continued to fluoresce until reacidification of these vesicles had occurred thus enabling the determination of reacidification kinetics (Atluri and Ryan, 2006; Granseth et al., 2006 Fig. S3). (**a**) SypHy fluorescence imaging in control cortical neuron. (**b**) SypHy fluorescence imaging in N-cadherin expressing (pre- and postsynaptically) cortical neuron. Monoexponential fits of reacidification kinetics are indicated. (**c**) Quantification of reacidification kinetics. Means ± SEM. n (cells) = 3 / 5.

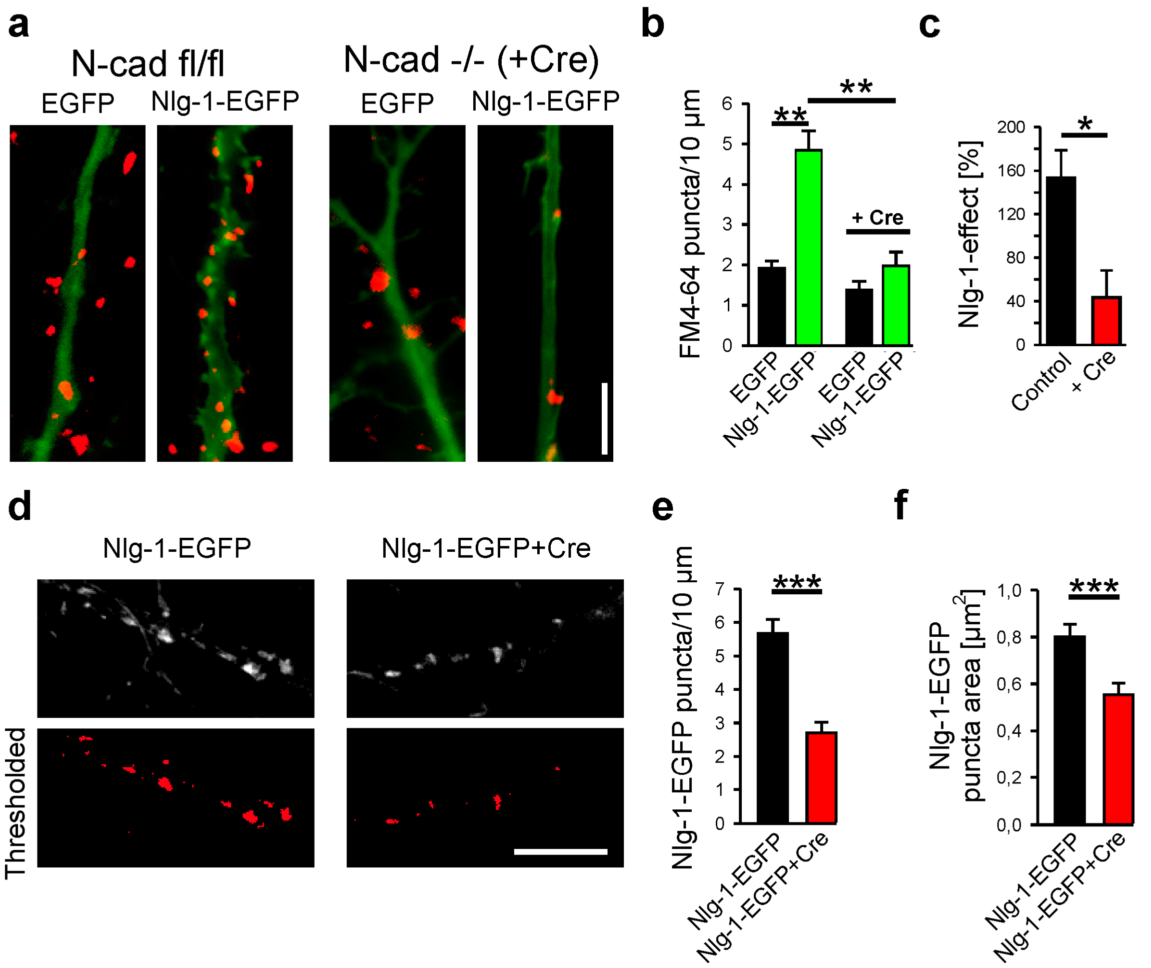


**Suppl. Figure 5. Neuroligin1-EGFP induced enhancement of vesicle cycling at mature synapses (at 12-14 DIV) required N-cadherin expression.** (**a**) Example overlay images of dendritic segments and FM4-64 puncta upon Nlg-1-EGFP expression and EGFP expression, respectively. +Cre: Co-expression of Cre to induce N-cadherin knockout in cultured neurons from floxed N-cadherin mice. Scale bar: 5 µm. (**b**) Quantification of dendritic density of FM4-64 puncta in the presence and upon knockout (+Cre) of N-cadherin. *n* (cells) = 14 / 6 / 12 / 12. (**c**) Effect size of Neuroligin-1-EGFP expression. (**d-f**) Neuroligin-1 clustering at mature synapses (at 14 DIV) depends on N-cadherin expression. (**d**) Example fluorescence images (upper panel) and thresholded Neuroligin1-EGFP puncta (lower panel). Scale bar: 5 µm. Quantification of dendritic density of Neuroligin1-EGFP puncta (**e**) and of Neuroligin1-EGFP puncta area (**f**). *n* (cells) = 30 / 33. Means ± SEM. One-way ANOVA with Holm-Sidak posthoc test (b, P<0.001), Student´s t-test (c, P=0.013; e, P<0.001; f, P<0.001).
